# MicroRNA-630 may confer favorable cisplatin-based chemotherapy and clinical outcomes in non-small cell lung cancer by targeting Bcl-2

**DOI:** 10.18632/oncotarget.24474

**Published:** 2018-02-09

**Authors:** Ming-Jenn Chen, De-Wei Wu, Gao-Chang Wang, Yao-Chen Wang, Chi-Yi Chen, Huei Lee

**Affiliations:** ^1^ Department of Surgery, Chi Mei Medical Center, Tainan, Taiwan, ROC; ^2^ Department of Sports Management, College of Leisure and Recreation Management, Chia Nan University of Pharmacy and Science, Tainan, Taiwan, ROC; ^3^ Graduate Institute of Cancer Biology and Drug Discovery, Taipei Medical University, Taipei, Taiwan, ROC; ^4^ Department of Internal Medicine, Chung Shan Medical University, Taichung, Taiwan, ROC; ^5^ Department of Surgery, Chung Shan Medical University, Taichung, Taiwan, ROC; ^6^ School of Medicine, Chung Shan Medical University, Taichung, Taiwan, ROC

**Keywords:** Micro-630, Bcl-2, cisplatin, chemotherapy, NSCLC

## Abstract

MicroRNA-630 (miR-630) plays dual roles in tumor progression in various human cancers. However, the role of miR-630 in chemoresistance and prognosis in non-small cell lung cancer (NSCLC) remains to be elucidated. This retrospective study enrolled 114 surgically resected patients with NSCLC who experienced tumor relapse and underwent cisplatin-based chemotherapy. The aim was to examine the possible association between miR-630 (and its targeting of Bcl-2 expression) and the response to cisplatin-based chemotherapy. Patients with tumors expressing low miR-630, high Bcl-2, and a combination of both were more likely than their counterparts to show unfavorable responses to cisplatin-based chemotherapy. Kaplan–Meier and Cox regression analysis indicated that low miR-630, high Bcl-2, and a combination of both may independently predict poor overall survival and short relapse-free survival in patients with NSCLC. Six types of NSCLC cells were collected to determine the inhibitory concentration of cisplatin yielding 50% viability (IC50) by the MTT assay. The IC50 value for cisplatin was negatively correlated with miR-630 expression levels among these cell types, except for A549 cells. Mechanistically, low miR-630 expression conferred cisplatin resistance and colony formation by de-targeting Bcl-2 in NSCLC cells. We therefore suggest that low miR-630, high Bcl-2, and a combination of both may potentially predict an unfavorable chemotherapeutic response and poor outcome in patients with NSCLC.

## INTRODUCTION

Chemotherapy remains an essential element of personalized care for patients with non-small cell lung cancer (NSCLC), despite the improvements in molecularly targeted and immunologically based therapies over the past two decades [1]. However, resistance to chemotherapy and tumor relapse have severe adverse impacts on patient outcomes. Therefore, treatments that can increase chemosensitivity are urgently needed for improving outcomes and the quality of life in patients with NSCLC.

One promising approach involves the use of microRNAs (miRs), which are biomarkers showing diagnostic and therapeutic potential in various human cancers, including NSCLC [2, 3] . One specific form, microRNA-630 (miR-630), is confirmed to play dual roles in tumor progression in a number of human cancers. For example, miR-630 acts as an oncogene, promoting tumor progression and consequently resulting in poor prognosis in colorectal, renal cell, gastric, and ovarian cancers [4–9]. Conversely, miR-630 has a tumor suppressor role, inhibiting tumor progression and metastasis in esophageal squamous cell, breast, and lung cancer [10–12]. Therefore, we suspected that the oncogenic and tumor suppressor roles of miR-630 in different types of cancers might dictate chemoresistance and chemosensitivity.

Surprisingly, miR-630 can reduce cell proliferation of A549 lung cancer cells and confer cisplatin resistance by increasing p27 expression [13]. However, in the same A549 cells, although miR-630 may inhibit cell proliferation by targeting CDC7 kinase, it may also maintain the apoptotic balance by targeting multiple modulators, such as PARP3, DDIT4, EP300, and p53 [14]. Upregulation of miR-630, which may target Bcl-2, and Bcl2l2 to modulate the apoptotic pathway, can occur following exposure of head and neck squamous cell carcinoma cells to cisplatin, which promotes the ATM-dependent phosphorylation of ΔNp63α [15]. Unfortunately, patients with NSCLC showed no cisplatin-based chemotherapeutic response to support findings of cisplatin sensitivity mediated by miR-630.

The present retrospective study collected 114 tumors that were surgically resected from NSCLC patients who had experienced tumor recurrence and had also received cisplatin-based chemotherapy. The aim was to evaluate expression of miR-630 and its targeting Bcl-2 by the real-time polymerase chain reaction (PCR) and immunohistochemistry and to analyze statistically the association of the expression of both molecules with the observed chemotherapeutic response. The prognostic value of miR-630 and Bcl-2 expression on overall survival and relapse free survival was assessed by Kaplan–Meier and Cox regression models. The miR-630 expression levels were also evaluated in a panel of lung cancer cells to select cells that showed high and a low expression of miR-630 for miR-630 manipulation and to verify whether miR-630 might modulate cisplatin sensitivity by targeting Bcl-2.

## RESULTS

### MiR-630 expression is associated with tumor stage and is correlated with Bcl-2 expression in patients with NSCLC

We evaluated 114 tumor tissues from patients with NSCLC for miR-630 expression by real-time PCR analysis. The median value of miR-630 expression levels in this study population was used as a cutoff point to divide patients into high-miR-630 and low-miR-630 subgroups. Bcl-2 expression was determined by immunohistochemistry and data collected from our previous studies [16]. Interestingly, the low-miR-630 subgroup more commonly had advanced rather than early stage tumors (57.7% for stage II and III vs. 37.2% for stage I, *P* = 0.034; Table 1), but miR-630 expression was not associated with any other clinical parameters including age, gender, smoking status, and tumor type (Table 1). In addition, no relationship was observed between Bcl-2 expression and the clinical parameters of this study population. Interestingly, the low miR-630 subgroup had a greater prevalence of high Bcl-2 expression than low Bcl-2 expression (68.4% vs. 31.6%, *P* = 0.005; Table 1). These results suggest that miR-630 expression in NSCLC might be negatively associated with tumor progression and negatively correlated with Bcl-2 expression.

**Table 1:** Relationships of miR-630 and Bcl-2 expression levels with clinico-pathological parameters, and the association between miR-630 and Bcl-2 expression levels in lung cancer patients

			miR-630			Bcl-2		
	No.		Low	High	*P*	Low	High	*P*
**Age**								
< 65	57		28(49.1)	29(50.9)	0.851	24 (42.1)	33 (57.9)	0.572
≥ 65	57		29(50.9)	28(49.1)		27 (47.4)	30 (52.6)	
**Gender**								
Female	38		20(52.6)	18(47.4)	0.691	14 (36.8)	24 (63.2)	0.231
Male	76		37(48.7)	39(51.3)		37 (48.7)	39 (51.3)	
**Smoking status**								
Nonsmokers		55	29(52.7)	26(47.3)	0.574	22 (40.0)	33 (60.0)	0.326
Smokers		59	28(47.5)	31(52.5)		29 (49.2)	30 (50.8)	
**Tumor type**								
Adenocarcinoma		56	25(44.6)	31(55.4)	0.261	22 (39.3)	34 (60.7)	0.250
Squamous		58	32(55.2)	26(44.8)		29 (50.0)	29 (50.0)	
**Stage of Cancer**								
1		43	16(37.2)	27(62.8)	0.034	21 (48.8)	22 (51.2)	0.493
2–3		71	41(57.7)	30(42.3)		30 (42.3)	41 (57.7)	
miR-630								
Low		57				18 (31.6)	39 (68.4)	0.005
High		57				33 (57.9)	24 (42.1)	

### Low miR-630 expression, high Bcl-2 expression, or a combination of both are associated with poor outcome in patients with NSCLC

**Figure 1:**
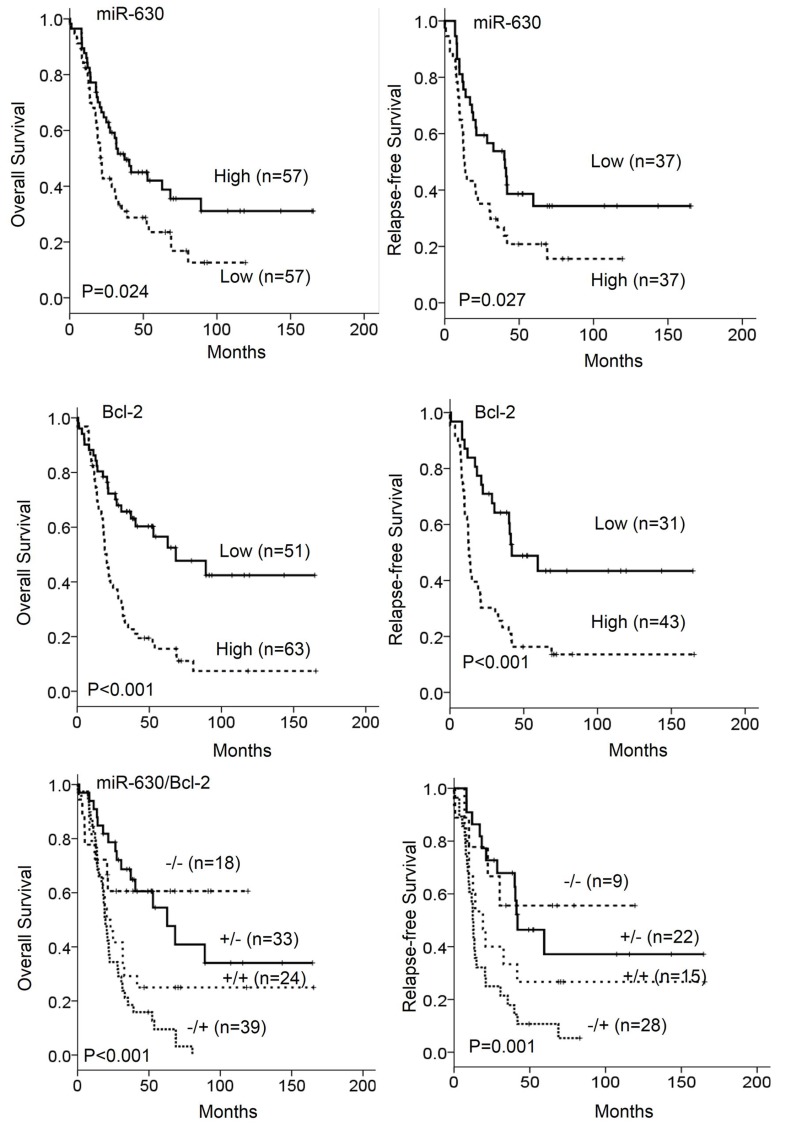
The expression of miR-630 and Bcl-2 in tumors was associated with overall survival (OS) and relapse free survival (RFS) in patients with lung cancer Patients with tumors showing low-miR-630 and high-Bcl-2 expression had poor outcomes.

We examined whether low miR-630 and high Bcl-2 expression could be associated with poor outcome in patients with NSCLC. Kaplan–Meier analysis indicated that patients with low miR-630 tumors and high Bcl-2 tumors had shorter overall survival (OS) and relapse-free survival (RFS) when compared with patients with high-miR-630 tumors and low Bcl-2 tumors (miR-630: *P* = 0.024 for OS; *P* = 0.027 for RFS, Figure 1 upper panel; Bcl-2: *P* < 0.001 for OS and RFS, Figure 1 median panel). In addition, Kaplan–Meier analysis indicated that patients with low miR-630 tumors with high Bcl-2 tumors had shortest overall survival (OS) and relapse-free survival (RFS) when compared with other groups (*P* < 0.001 for OS and *P* = 0.001 for RFS, Figure 1 lower panel). Cox-regression further indicated that miR-630 and Bcl-2 expression had prognostic significance for OS and RFS in this study population (Table 2). The hazard ratio (HR) values for low-miR-630 tumors for OS and RFS were 1.65 and 1.90 when high-miR-630 tumors were used as a reference (95% CI, 1.02–2.64, *P* = 0.037 for OS; 95% CI, 1.06–3.42, *P* = 0.031 for RFS; Table 2). The HR values of high Bcl-2 tumors for OS and RFS were 2.68 and 2.84 when low Bcl-2 tumors were as a reference (95% CI, 1.62–4.36, *P* < 0.001 for OS, 95% CI, 1.51–5.33, *P* = 0.001 for RFS; Table 2). Moreover, the HR value was higher for low miR-630/high Bcl-2 tumors than for tumors with low miR-630/low Bcl-2 or high miR-630/high Bcl-2 expression when compared with high miR-630/low Bcl-2 tumors (HR, 2.98 vs. 1.65 vs. 2.68, *P* < 0.001 for OS, 3.45 vs. 1.90 vs. 2.84, *P* = 0.001 for RFS; Table 2). These results suggest that low miR-630 expression, high Bcl-2 expression, or a combination of both may independently predict poor outcome in patients with NSCLC.

**Table 2:** Cox regression analysis for the prognostic value of miR-630 and Bcl-2 expression levels on OS and RFS in lung cancer patients

	OS				RFS			
	Case No.	HR^*^	95%CI	*P*	Case No.	HR^*^	95%CI	*P*
**miR-630**								
High	57	1			37	1		
Low	57	1.65	1.02-2.64	0.037	37	1.90	1.06-3.42	0.031
**Bcl-2**								
Low	51	1			31	1		
High	63	2.68	1.62-4.36	<0.001	43	2.84	1.51-5.33	0.001
**miR-630/Bcl-2**								
+/−	33	1			22	1		
−/−	18	0.84	0.33-2.10	0.704	9	1.06	0.32-3.48	0.927
+/+	24	1.88	0.93-3.77	0.077	15	2.15	0.89-5.13	0.085
−/+	39	2.98	1.63-5.44	<0.001	28	3.45	1.61-7.35	0.001

OS: overall survival; HR: Hazard ratio; RFS: relapse free survival.

^*^HR for all cases was adjusted by age, gender, smoking status, tumor type and stage.

### Low miR-630 and high Bcl-2 expression are associated with an unfavorable response to cisplatin-based chemotherapy in patients with NSCLC

We next examined the possibility that low miR-630 and high Bcl-2 expression could be associated with an unfavorable chemotherapeutic response in patients with NSCLC. Of the 114 enrolled patients, 74 were available for assessing the correlation of miR-630 and Bcl-2 expression, respectively, with chemotherapeutic response. The patients with low miR-630 and high Blc-2 expressing tumors were more likely to have an unfavorable response to cisplatin-based chemotherapy when compared with patients with high miR-630 and low Bcl-2 expressing tumors (miR-630: 67.4% vs. 30.3%, *P* = 0.003; Bcl-2: 67.4% vs. 29.0%, *P* = 0.001; Table 3). These results suggest that low miR-630 and high Bcl-2 expression may predict an unfavorable response to chemotherapy in patients with NSCLC.

**Table 3:** The association of miR-630 and Bcl-2 expression levels with the response to cisplatin-based therapy in lung cancer patients

		The response to cisplatin therapy		
Variables	Patient No.	Unfavorable (%)	Favorable (%)	*P*
**miR-630**				
Low	42	28 (66.7)	14 (33.3)	0.003
High	32	10 (31.3)	22 (68.8)	
**Bcl-2**				
Low	31	9 (29.0)	22 (71.0)	0.001
High	43	29 (67.4)	14 (32.6)	

Complete response (CR): a complete disappearance of all the tumors; partial response (PR): a decrease in size or number of the tumor lesions by 50% or more; progressive disease (PD): at least 25% increase in size or number of the tumor lesions; and stable disease (SD): neither sufficient shrinkage to qualify for partial response nor sufficient increase to qualify for progressive disease. Therefore, the favorable response (CR and PR) is the decrease of tumor size at least 50% or more.

### Low miR-630 expression may confer cisplatin resistance in NSCLC cells

**Figure 2:**
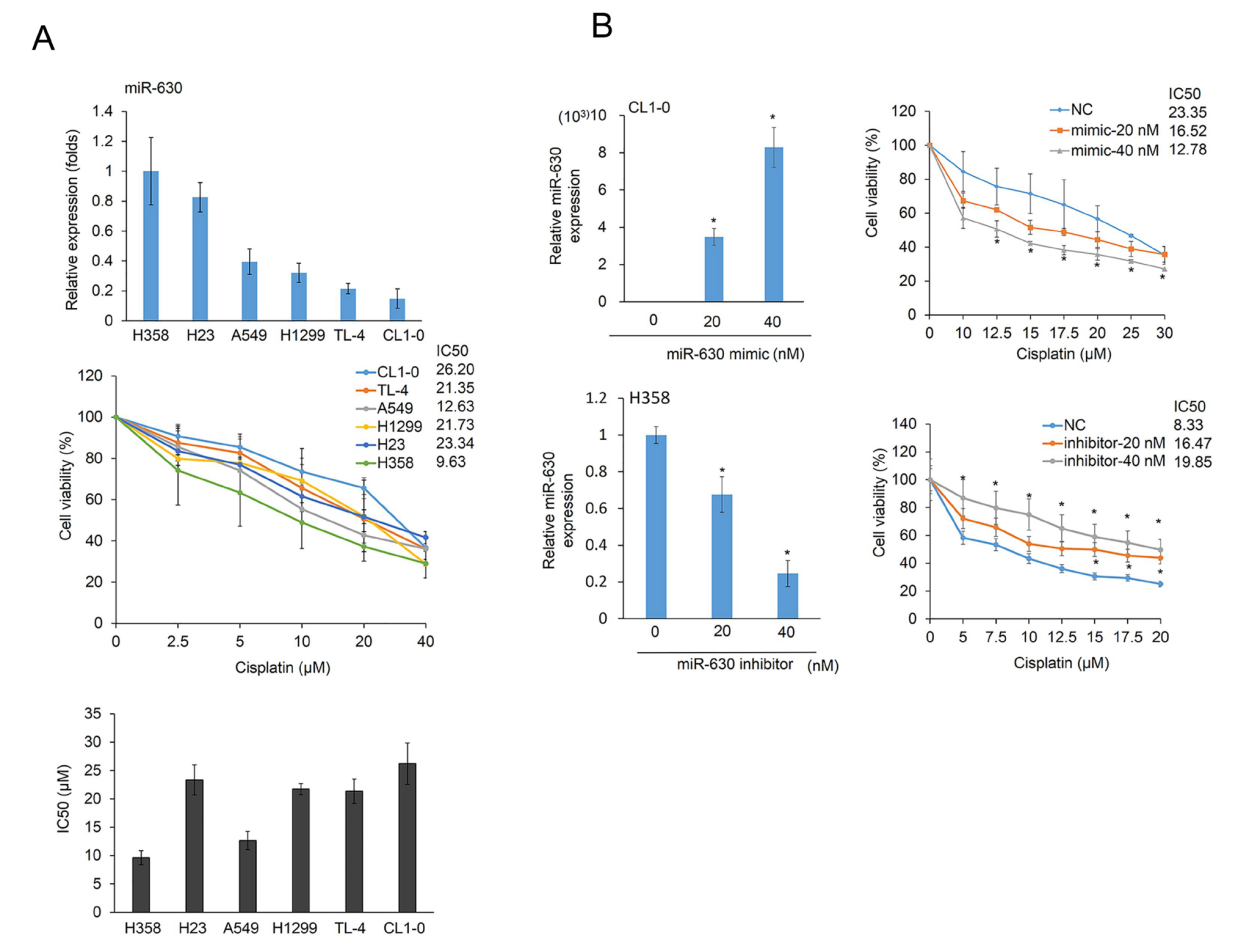
MiR-630 expression levels are associated with cisplatin resistance (**A**) Six lung adenocarcinoma cells were treated with five concentrations of cisplatin. After 48 h, the dose-response curves, determined by the MTT assay, were used to calculate the IC50 values of these cells. MiR-630 expression of these cells was evaluated by real-time PCR. (**B**) A miR-630 inhibitor was transfected into H358 cells. MiR-630 mimics were transfected into low miR-630 expressing CL1-0 cells. After 24 h, the cells were treated with various concentrations of cisplatin to calculate the IC50 values. NC: nonspecific control. *P* value was calculated by the Student’s *t*-test. The significant differences in experimental groups were compared to vehicle or indicated treatment (^*^P < 0.05). N.s.: Non-significant.

Six NSCLC cell types were collected to evaluate miR-630 expression levels by real-time PCR. The highest miR-630 expression was observed in H358 cells, followed by H23, A549, H1299, TL4, and CL1-0 cells (Figure 2A upper panel). The MTT assay was performed to calculate the inhibitory concentration of cisplatin yielding 50% viability (IC50) in these six cell types. The highest IC50 value for cisplatin was observed in CL1-0 cells, followed by TL4, H1299, H23, and A549 cells (Figure 2A low panel). In general, miR-630 expression levels were negatively associated with the IC50 value for cisplatin in these cell types, except in A549 cells. The low miR-630 expressing CL1-0 and the high miR-630 expressing H358 cells were selected for further miR-630 manipulation using a miR-630 mimic and inhibitor. The expression levels of miR-630 in both cell types were increased and decreased by miR-630 manipulation in a dose-dependent manner (Figure 2B left panel). The MTT assay indicated that the IC50 value for cisplatin in CL1-0 cells was markedly decreased by transfection of a miR-630 mimic; conversely, the IC50 value for cisplatin in H358 cells was significantly increased by treatment with a miR-630 inhibitor (Figure 2B right panel). These results indicated that low miR-630 expression may confer cisplatin resistance in NSCLC cells.

### Low miR-630 expression may confer cisplatin resistance and promote colony formation by de-targeting Bcl-2

**Figure 3:**
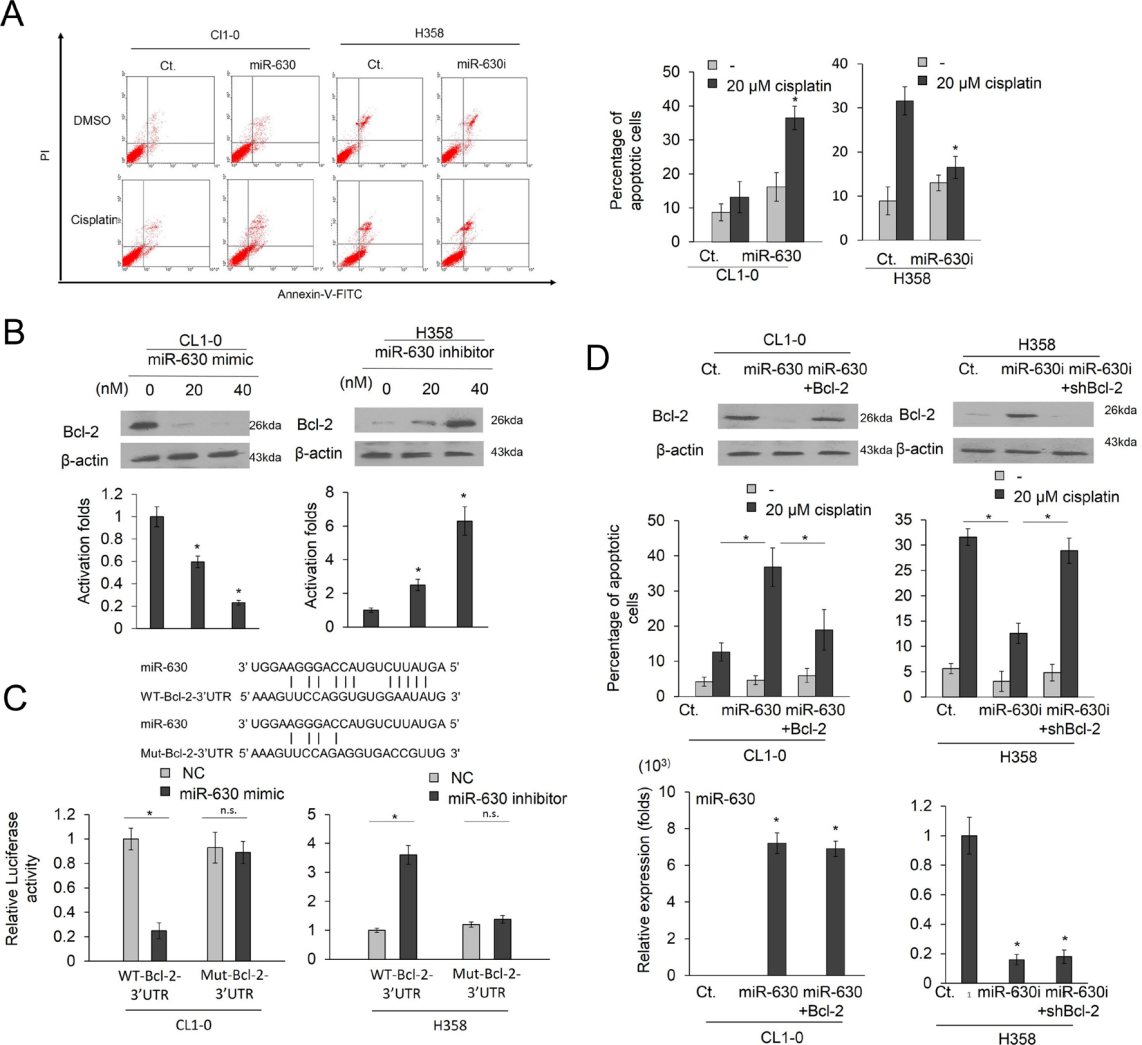
Low miR-630 expression may confer cisplatin resistance and promote colony formation by de-targeting Bcl-2 (**A**) MiR-630 inhibitor were transfected into H358 cells. MiR-630 mimics were transfected into CL1-0 cells. After 24 h, these cells were treated with 0.1% DMSO or 20 μM cisplatin for 48 h and then subjected to annexin-V and PI staining, followed by flow cytometry analysis. The percentages of apoptotic cells included with the annexin V+/PI- population plus annexin-V+/PI+ are summarized. (**B**) MiR-630 inhibitor were transfected into H358 cells. MiR-630 mimics were transfected into CL1-0 cells. After 48 h, the cells lysates were evaluated for the expression of Bcl-2 expression by real-time PCR and western blotting. (**C**) Top: The miR-630 binding sequence of wild-type (WT) or mutant (Mut) Bcl-2-3′-UTR were synthesized and ligated with pmiR-REPORT miRNA Expression Reporter Vector. Bottom: CL1-0 and H358 cells were transfected with miR-630 mimic, miR-nonspecific control, pMIR-Reporter luciferase vector, including 3′-UTR of Bcl-2 (with WT or mutant miR-630 response element) and β- galactosidase plasmid. In all experiments, the relative level in the NC controls was arbitrarily assigned as 1. (**D**) CL1-0 cells were transfected with the indicated combination of miR-630 mimics and Bcl-2, overexpression plasmids for 24 h. H358 cells were transfected with the indicated combination of miR-630 inhibitor and shBcl-2 for 24 h. These cells were treated with 0.1% DMSO or 20 μM of cisplatin for 48 h and were subjected to annexin-V and PI staining, followed by a flow cytometry analysis. The percentages of the apoptotic cells in the annexin V+/PI- population plus annexin-V+/PI+ are summarized. *P* value was calculated by the Student’s *t*-test. The significant differences in experimental groups were compared to vehicle or indicated treatment (^*^P < 0.05). N.s.: Non-significant.

Representative profiles of Annexin-V/PI staining are presented in Figure 3A (left panel). Transfection with a miR-630 mimic markedly increased the percentage of apoptotic cells induced by cisplatin in CL1-0 cells (Figure 3A middle panel). By contrast, the induction of apoptotic cells by cisplatin was almost completely eliminated in H358 cells transfected with a miR-630 inhibitor when compared with control cells (Figure 3A right panel). Representative cisplatin-induced colony formation in both cell types following miR-630 mimic and inhibitor treatments are presented in Figure 3B (upper panel). The findings of Annexin-V/PI staining and suggested that miR-630 may trigger cisplatin-induced apoptosis and reduction in colony formation.

We hypothesized that the induction of cisplatin resistance by low miR-630 could occur through a de-targeting of Bcl-2. Western blotting indicated that Bcl-2 expression in CL1-0 cells was almost completely eliminated by transfection with a miR-630 mimic, but its expression in H358 cells was markedly elevated by miR-630 inhibitor treatment. Real-time PCR showed that the decrease and increase in Bcl-2 mRNA expression by the miR-630 mimic and inhibitor in both cell types were consistent with the changes in Bcl-2 protein expression (Figure 3B). In addition, Bcl-2 expression levels were positively associated with the IC50 value for cisplatin but were negatively corrected with miR-630 expression in these cell types, except in A549 cells (Supplementary Figure 1). To obtain further direct evidence that Bcl-2 is a target of miR-630, the miR-630 binding sequence of wild-type (WT) or mutant (Mut) Bcl-2-3′-UTR, as shown in Figure 3C, were synthesized and ligated with a pmiR-REPORT miRNA Expression Reporter Vector (Ambion, USA), and consequently transfected into CL1-0 and H358 cells, respectively. The luciferase reporter assay showed that the reporter activity of WT-Bcl-2-3′-UTR was markedly reduced by miR-630 mimic in CL1-0 cells, but almost was unchanged in the reporter activity of Mut-Bcl-2-3′-UTR when compared with miR-nonspecific control (NC) cells (Figure 3C). Conversely, the luciferase reporter assay showed that the reporter activity of WT-Bcl-2-3′-UTR was markedly increased by transfecting miR-630 inhibitor into H358 cells, but was nearly completely unchanged in the reporter activity of Mut-Bcl-2-3′-UTR when compared with miR-nonspecific control (NC) cells (Figure 3C). These results suggest that miR-630 may modulate Bcl-2 expression in lung cancer cells by directly targeting its 3′-UTR. CL1-0 cells were treated with a miR-630 mimic and/or Bcl-2 expression plasmid, whereas H358 cells were transfected with a miR-630 inhibitor and/or a small hairpin RNA (sh)Bcl-2. The Bcl-2 expression was decreased in the CL1-0 and increased in the H358 cells by miR-630 manipulation, but was restored by transfection of a Bcl-2 expression plasmid or shBcl-2, respectively (Figure 3D upper panel). The expression levels of miR-630 in miR-630-overexpressing CL1-0 and miR-630-silencing H358 cells were unchanged by Bcl-2 manipulation (Figure 3D). The percentage of apoptotic cells induced by cisplatin was elevated by miR-630 mimic transfection in CL1-0 cells, but this increase in the percentage of apoptotic cells was reversed by transfection of a Bcl-2 expression plasmid (Figure 3D lower left panel). Conversely, the percentage of apoptotic cells induced by cisplatin was significantly reduced in H358 cells by transfection of a miR-630 inhibitor than in H358 cells by transfection of a non-specific inhibitor (NC). However, the percentage of apoptotic cells induced by cisplatin was significantly increased in H358 cells by transfection of a miR-630 inhibitor plus shBcl-2 as compared with H358 cells by transfection of a miR-630 inhibitor. (Figure 3D lower right panel). These results clearly indicate that low miR-630 may confer cisplatin resistance and promote colony formation by modulating the apoptotic pathway due to a de-targeting of Bcl-2.

## DISCUSSION

We have provided evidence that patients with NSCLC tumors showing low expression of miR-630, high expression of Bcl-2, or a combination of both may have an unfavorable response to cisplatin-based chemotherapy and a poor outcome. This is the first study to report an association of expression of miR-630, Bcl-2, or a combination, and the chemotherapeutic response and prognosis in patients with NSCLC.

In cell model experiments, low miR-630 expression may promote colony formation and cisplatin resistance by a de-targeting of Bcl-2. This result was consistent with previous studies indicating that miR-630 may suppress cell proliferation and induce apoptosis by targeting Bcl-2 [15, 17]. However, a contradictory finding of cisplatin sensitivity mediated by miR-630 was reported in A549 lung cancer cells [13, 14].

Cao *et al.* (2014) showed that miR-630 played dual roles in A549 cell apoptosis as it targets the cell proliferation regulator CDC7 kinase and the apoptotic modulator EP300 to promote p53 protein instability [14]. However, the IC50 value for cisplatin in p53 wild-type A549 cells was markedly elevated following transfection with a miR-630 mimic and reduced following transfection with an inhibitor (Supplementary Figure 1), which supported the findings of Kroemer’s group [13]. This finding indicates that miR-630 may confer cisplatin resistance in A549 cells. These results showed that the effects of miR-630 on cisplatin resistance differed in A549 cells when compared to CL1-0 and H358 cells. As shown in Figure 1A, miR-630 expression levels were relatively lower in p53-WT TL-4 cells than in p53-WT A549 cells. As expected, the IC50 value of TL-4 cells was greater than that of A549 cells (21.35 vs. 12.63). Real-time PCR analysis indicated that the expression of Bcl-2 was relatively higher in TL-4 cells than in A549 cells (Supplementary Figure 2). Mechanistic studies further confirmed that low miR-630 conferred cisplatin resistance in TL-4 cells via de-targeting Bcl-2 (Supplementary Figure 3). These results obtained from p53-WT cells were similar with the observations of p53-mutated cells (Figures 2 and 3). These results strongly suggested that low miR-630 may confer cisplatin resistance in p53-WT and -mutated lung cancer cells via de-targeting Bcl-2.

In summary, patients with tumors with low miR-630 expression, high Bcl-2 expression, or a combination of both, show responses to cisplatin-based chemotherapy that strongly support the mechanism of action suggested by cell models; namely, that low miR-630 expression may confer cisplatin resistance by a de-targeting of Bcl-2. Therefore, we suggest that tumors showing a combination of low miR-630 expression and high Bcl-2 expression might be predictors of poor chemotherapeutic response and unfavorable prognosis in patients with NSCLC.

## MATERIAL AND METHODS

### Chemicals and antibodies

Cisplatin were obtained from Selleckchem (Houston, TX, USA). Anti-Bcl-2 was obtained from Genetex (Irvine CA, USA). All other antibodies were purchased from Santa Cruz Biotechnology (Dallas, TX, USA).

### Human study subjects

The study included 114 patients who underwent resected at the Department of Chest Surgery, Taichung Veteran General Hospital, Taichung, Taiwan, between June 1994 and December 2006. The tumor type and stage of each specimen was histologically determined according to the World Health Organization’s (WHO) classification system. The median follow-up time was 24.3 months (range from 1 to 165.3 months) and the end of the follow-up period was Dec. 2007. Seventy-four of 114 patients were received adjuvant cisplatin-based chemotherapy. As shown in the Supplementary Table 1, two patients received single-agent cisplatin, but 74 patients received cisplatin plus etoposide, gemzar, taxol, or taxotere. The IRB protocol 201305020 was approved by Taipei Medical University.

### Cell lines

The lung cancer cells were obtained from the American Type Culture Collection (ATCC) and cultured as described.

### Plasmid constructs and transfection

The Flag-Bcl-2 (#18003) overexpression plasmid was purchased from Addgene (Cambridge, MA, USA). Bcl-2 (TRCN0000040071) were purchased from National RNAi Core Facility, Academia Sinica, Taiwan. These plasmids were transiently transfected into lung cancer cells (1 × 10^6^) using the Turbofect reagent (Formentas, Glen Burnie, MD, USA). After 48 h, cells were harvested to assay in subsequent experiments.

### MiR-630 precursor and inhibitor transfection

Cells were grown to confluence in 6-well plates. The miR-630 precursor (40 nM) (Ambion, Foster city, CA, USA), miR-630 inhibitor (80 nM) (Ambion) and negative control (Ambion) cells were transfected using Lipofectamine 3000 transfection reagent (Invitrogen, Foster city, CA, USA) according to the manufacturer’s protocol. Transfection efficiency was evaluated by the real-time PCR.

### MTT cytotoxicity assay, Annexin V/PI staining

MTT cytotoxicity assay and Annexin V/PI staining were performed as described in a previous report [18].

### Real-time quantitative RT-PCR analysis

DNase I-treated total RNA (10 ng) was subjected to microRNA polymerase chain reaction (PCR) analysis with the TaqMan^®^ miRNA Reverse Transcription Kit Life technologies, Foster city, CA, USA), miRNA Assays (Life technologies, Foster city, CA, USA), and a Real-Time Thermocycler 7500 (Life technologies, Foster city, CA, USA). RNU6B was used as the small RNA reference housekeeping gene. The following primer sequences were used for amplification of the Bcl-2 gene: the forward primer, 5′- CTGTGGATGACTGAGTACC -3′ and the reverse primer, 5′- CAGCCAGGAGAAATCAAAC -3′. The miR-630 and Bcl-2 mRNA levels in lung tumors that were higher than the median value were defined as “high”, while levels lower than the median value were defined as “low”.

### Immunohistochemistry (IHC)

Bcl-2 immunohistochemical staining scores were defined as described previously [16] and the intensities of signals were evaluated independently by three observers. Immunostaining scores were defined as the cell staining intensity (0 = nil; 1 = weak; 2 =moderate; and 3 = strong) multiplied by the percentage of labelled cells (0–100%), leading to scores from 0 to 300. A score of over 150 was rated as “high” immunostaining, while a score of less than 150 was rated as “low”.

### Western blotting

Western blotting was performed as described in a previous report [19].

### Statistical analysis

Statistical analysis was performed using the SPSS statistical software (Version 18.0; Chicago, IL, USA). For cell model experiments, *P* value was calculated by the Student’s *t*-test.

## SUPPLEMENTARY MATERIALS FIGURES AND TABLE



## References

[1] Hellmann MD, Li BT, Chaft JE, Kris MG (2016). Chemotherapy remains an essential element of personalized care for persons with lung cancers. Ann Oncol.

[2] Del Vescovo V, Grasso M, Barbareschi M, Denti MA (2014). MicroRNAs as lung cancer biomarkers. World J Clin Oncol.

[3] Shen J, Jiang F (2012). Applications of MicroRNAs in the Diagnosis and Prognosis of Lung Cancer. Expert Opin Med Diagn.

[4] Zhao JJ, Chen PJ, Duan RQ, Li KJ, Wang YZ, Li Y (2016). miR-630 functions as a tumor oncogene in renal cell carcinoma. Arch Med Sci.

[5] Chu D, Zheng J, Li J, Li Y, Zhang J, Zhao Q, Wang W, Ji G (2014). MicroRNA-630 is a prognostic marker for patients with colorectal cancer. Tumour Biol.

[6] Chu D, Zhao Z, Li Y, Li J, Zheng J, Wang W, Zhao Q, Ji G (2014). Increased microRNA-630 expression in gastric cancer is associated with poor overall survival. PLoS One.

[7] Gu L, Li H, Chen L, Ma X, Gao Y, Li X, Zhang Y, Fan Y, Zhang X (2015). MicroRNAs as prognostic molecular signatures in renal cell carcinoma: a systematic review and meta-analysis. Oncotarget.

[8] Zhao JJ, Chen PJ, Duan RQ, Li KJ, Wang YZ, Li Y (2014). Up-regulation of miR-630 in clear cell renal cell carcinoma is associated with lower overall survival. Int J Clin Exp Pathol.

[9] Zou YT, Gao JY, Wang HL, Wang Y, Wang H, Li PL (2015). Downregulation of microRNA-630 inhibits cell proliferation and invasion and enhances chemosensitivity in human ovarian carcinoma. Genet Mol Res.

[10] Jin L, Yi J, Gao Y, Han S, He Z, Chen L, Song H (2016). MiR-630 inhibits invasion and metastasis in esophageal squamous cell carcinoma. Acta Biochim Biophys Sin (Shanghai).

[11] Zhou CX, Wang CL, Yu AL, Wang QY, Zhan MN, Tang J, Gong XF, Yin QQ, He M, He JR, Chen GQ, Zhao Q (2016). MiR-630 suppresses breast cancer progression by targeting metadherin. Oncotarget.

[12] Song YF, Hong JF, Liu DL, Lin QA, Lan XP, Lai GX (2015). miR-630 targets LMO3 to regulate cell growth and metastasis in lung cancer. Am J Transl Res.

[13] Galluzzi L, Morselli E, Vitale I, Kepp O, Senovilla L, Criollo A, Servant N, Paccard C, Hupé P, Robert T, Ripoche H, Lazar V, Harel-Bellan A (2010). miR-181a and miR-630 regulate cisplatin-induced cancer cell death. Cancer Res.

[14] Cao JX, Lu Y, Qi JJ, An GS, Mao ZB, Jia HT, Li SY, Ni JH (2014). MiR-630 inhibits proliferation by targeting CDC7 kinase, but maintains the apoptotic balance by targeting multiple modulators in human lung cancer A549 cells. Cell Death Dis.

[15] Huang Y, Chuang A, Hao H, Talbot C, Sen T, Trink B, Sidransky D, Ratovitski E (2011). Phospho-ΔNp63α is a key regulator of the cisplatin-induced microRNAome in cancer cells. Cell Death Differ.

[16] Wu DW, Wu TC, Wu JY, Cheng YW, Chen YC, Lee MC, Chen CY, Lee H (2014). Phosphorylation of paxillin confers cisplatin resistance in non-small cell lung cancer via activating ERK-mediated Bcl-2 expression. Oncogene.

[17] Farhana L, Dawson MI, Murshed F, Das JK, Rishi AK, Fontana JA (2013). Upregulation of miR-150* and miR-630 induces apoptosis in pancreatic cancer cells by targeting IGF-1R. PLoS One.

[18] Wu DW, Chen TC, Huang HS, Lee H (2016). TC-N19, a novel dual inhibitor of EGFR and cMET, efficiently overcomes EGFR-TKI resistance in non-small-cell lung cancer cells. Cell Death Dis.

[19] Song S, Honjo S, Jin J, Chang SS, Scott AW, Chen Q, Kalhor N, Correa AM, Hofstetter WL, Albarracin CT, Wu TT, Johnson RL, Hung MC (2015). The Hippo Coactivator YAP1 Mediates EGFR Overexpression and Confers Chemoresistance in Esophageal Cancer. Clin Cancer Res.

