# Supplementary material for: MicroRNA-630 may confer favorable cisplatin-based chemotherapy and clinical outcomes in non-small cell lung cancer by targeting Bcl-2
Source: Oncotarget. 2018 Feb 9;9(17):13758–67. doi: 10.18632/oncotarget.24474 (PMC5862613; doi:10.18632/oncotarget.24474)
Supplement: Supplementary file 1 [file oncotarget-09-13758-s001.pdf]

MicroRNA-630 may confer favorable cisplatin-based chemotherapy and clinical outcomes in non-small cell lung cancer by targeting Bcl-2

SUPPLEMENTARY MATERIALS

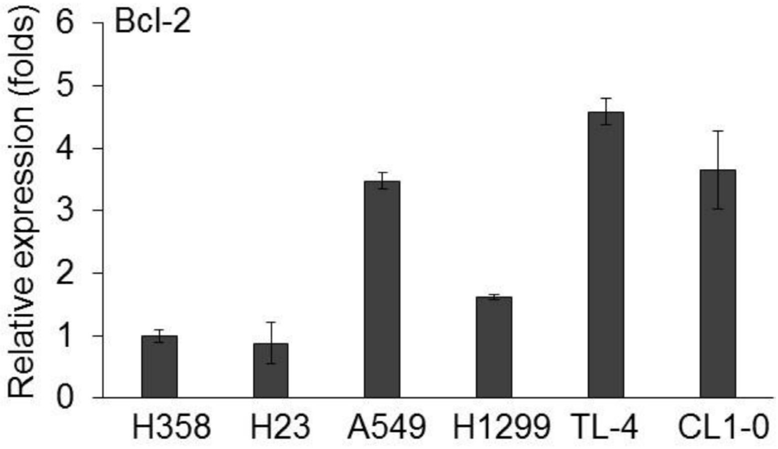

Supplementary Figure 1: Bcl-2 expression levels are associated with cisplatin resistance. Bcl-2 expression of these cells was evaluated by real-time PCR.

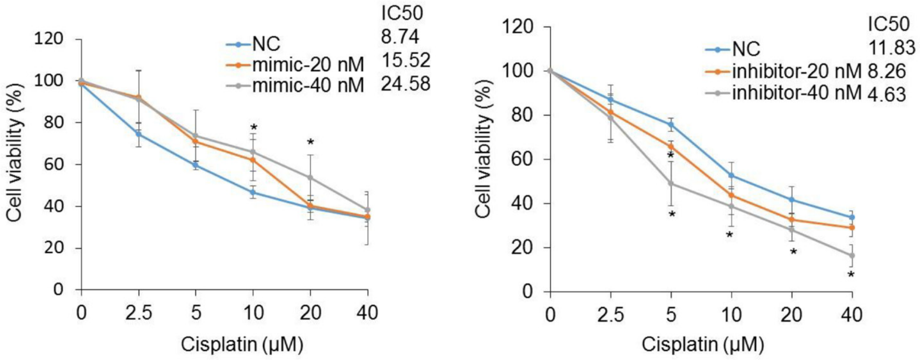

Supplementary Figure 2: The IC50 value for cisplatin in p53 wild-type A549 cells was markedly elevated following transfection with a miR-630 mimic and reduced following transfection with an inhibitor. MiR-630 inhibitor and mimics were transfected into A549 cells. After 24 h, the cells were treated with various concentrations of cisplatin to calculate the IC50 values. NC: nonspecific control. The significant differences in experimental groups were compared to NC control ($^{*}P < 0.05$).

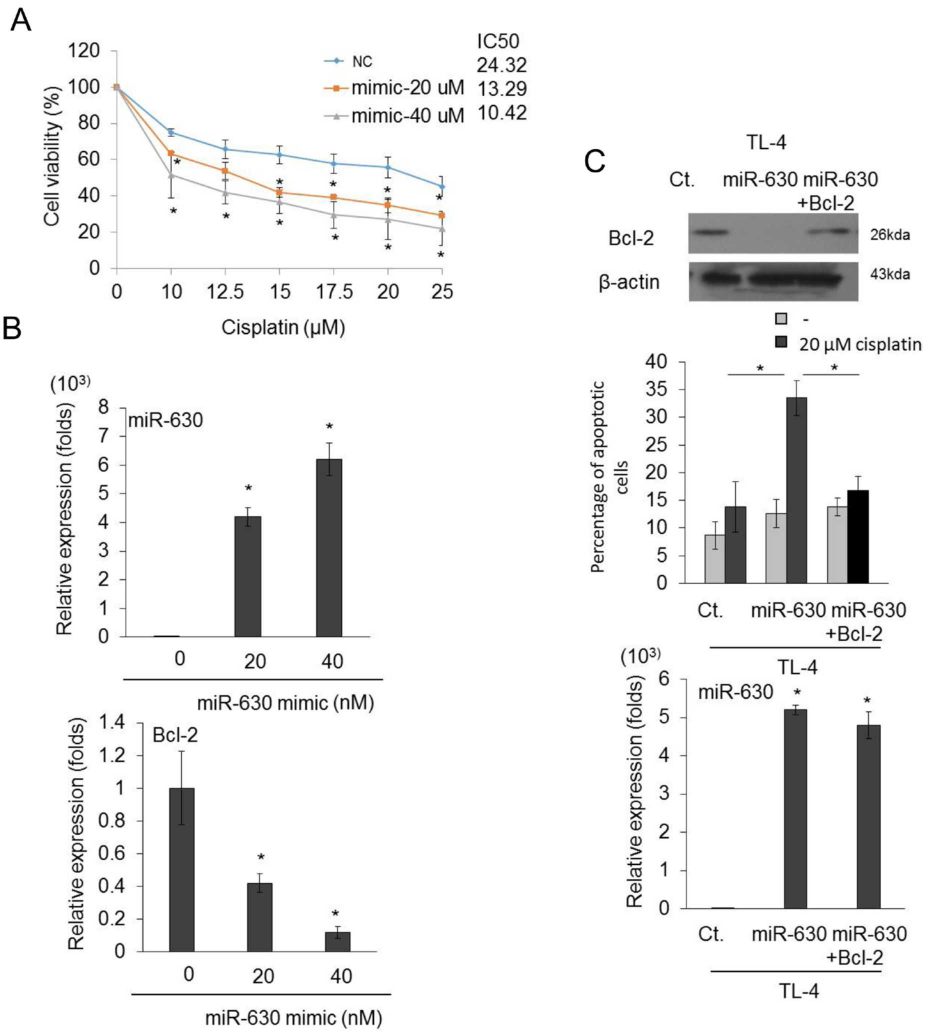

Supplementary Figure 3: MiR-630 expression levels are associated with cisplatin resistance in TL-4 cells. (**A**) MiR-630 mimics were transfected into low miR-630 expressing TL-4 cells. After 24 h, the cells were treated with various concentrations of cisplatin to calculate the IC50 values. NC: nonspecific control. (**B**) MiR-630 mimics were transfected into TL-4 cells. After 48 h, the cells lysates were evaluated for the expression of Bcl-2 and miR-630 expression by real-time PCR. (**C**) TL-4 cells were transfected with the indicated combination of miR-630 mimics and Bcl-2, overexpression plasmids for 24 h. These cells were treated with 0.1% DMSO or 20 μM of cisplatin for 48 h and were subjected to annexin-V and PI staining, followed by a flow cytometry analysis. The percentages of the apoptotic cells in the annexin V+/PI− population plus annexin-V+/PI+ are summarized. *P* value was calculated by the Student's t-test. The significant differences in experimental groups were compared to NC control or indicated treatment ($^*P < 0.05$). N.s.: Non-significant.

Supplementary Table 1: The anti-cancer agents used in lung cancer patients who have received cisplatin-based chemotherapy

Anti-cancer agents	Patient No. (%)
Cisplatin + etoposide	1 (1)
Cisplatin + gemzar	49 (66)
Cisplatin + taxol	20 (27)
Cisplatin + taxotere	2 (3)
Cisplatin	2 (3)

Seventy-four out of 114 patients have received cisplatin-based chemotherapy. Cisplatin and/or combination of etoposide, gemzar, toxol, or taxotere were used to treat with patients.
